# Performance Evaluation of a Sandwich-Structured CNT/Graphene–TPU Nanofiber Strain Sensor for Wearable Deformation Monitoring

**DOI:** 10.3390/mi17080959

**Published:** 2026-08-14

**Authors:** Heng Su, Shengbin Cao, Xue Zhang, Zeyu Liu, Xiaosong Liu

**Affiliations:** 1 School of Art and Design, Shanghai Dianji University, Shanghai 201306, China; 2 School of Materials Science and Engineering, Shanghai Dianji University, Shanghai 201306, China; 3 College of Materials Science and Engineering, Donghua University, Shanghai 201620, China; 4 School of Energy and Power Engineering, University of Shanghai for Science and Technology, Shanghai 200093, China; 5 Oxford Suzhou Centre for Advanced Research, Suzhou 215123, China

**Keywords:** TPU nanofibers, carbon nanotubes, graphene, flexible strain sensor, sandwich structure

## Abstract

Flexible strain sensors require both a responsive conductive network and a mechanically compliant supporting structure. In this study, a sandwich-structured carbon nanotube (CNT)/graphene–thermoplastic polyurethane (TPU) nanofiber strain sensor was fabricated by electrospinning, spray-coating, pre-stretching, and hot-pressing. Field-emission scanning electron microscopy was used to examine the nanofiber and coated-fiber morphology, while energy-dispersive X-ray spectroscopy was used only to describe elemental distribution. The electrical response was quantitatively evaluated under tensile deformation. The sensor exhibited piecewise gauge factors of approximately 47.3, 269.6, and 613.8 over strain ranges of 0–20%, 20–45%, and 45–60%, respectively. The response and recovery times were approximately 120 and 180 ms, and the electrical response remained observable over 5000 loading–unloading cycles at 20% strain. Qualitative demonstrations involving finger-bending, elbow-bending, pulse, grasping, walking, and repeated pressing produced distinguishable resistance-time patterns. These results indicate the potential of the CNT/graphene–TPU device for wearable deformation monitoring.

## 1. Introduction

With the rapid development of the Internet of Things, artificial intelligence, wearable electronics, and intelligent medical monitoring, flexible strain sensors, as key devices for sensing external mechanical stimuli and converting them into electrical signals [1,2,3,4,5,6], have shown broad application prospects in fields such as human motion monitoring, health management, electronic skin, soft robotics, and human–computer interaction [7,8,9,10,11]. Compared with traditional rigid strain sensors, flexible strain sensors can conform to human skin, joints, and clothing surfaces, enabling continuous monitoring under complex curvatures and dynamic deformation conditions, making them more suitable for wearable applications [12,13,14,15]. However, human motion and physiological signals exhibit characteristics such as large amplitude variations, rapid frequency changes, and variable action positions, requiring sensors to possess high sensitivity, a wide detection range, fast response, low hysteresis, good cycling stability, and comfortable wearability [16,17,18,19,20].

From the perspective of sensing mechanism, flexible strain sensors usually rely on changes in the contact area of conductive networks under external deformation, the reconstruction of conductive pathways, and variations in tunneling distance to achieve resistance-signal output [21,22,23]. Compared with capacitive, piezoelectric, and triboelectric sensors, piezoresistive strain sensors offer the advantages of simple structure, convenient signal readout, low fabrication cost, and easy miniaturization and array integration [24,25,26,27], and are therefore widely used in wearable deformation monitoring.

The selection of flexible substrate materials directly affects the mechanical adaptability and long-term stability of sensors. Thermoplastic polyurethane (TPU) exhibits excellent flexibility, wear resistance, stretchability, and spinnability [28,29,30]. TPU nanofiber membranes prepared by electrospinning possess a three-dimensional porous structure, high specific surface area, and good compressive resilience, which can provide abundant attachment sites and stress-buffering space for conductive fillers [31,32,33]. However, the thermal stability of TPU at higher temperatures may be limited and may be affected by hydrolysis and ultraviolet (UV) aging, thereby limiting its long-term use in harsh environments. Conductive fillers are another key factor determining piezoresistive performance. Carbon nanotubes (CNTs) exhibit excellent electrical conductivity, mechanical strength, and aspect ratio, and can form one-dimensional bridging conductive pathways on polymer-matrix surfaces [34,35]. Graphene has a two-dimensional layered structure, high carrier mobility, and large specific surface area, which can provide planar conductive contacts and multi-point electron-transport channels [36,37,38]. Upon compounding CNTs with graphene, CNTs can serve as flexible conductive bridge between layers, while graphene can provide surface contact platforms and local conductive islands, constructing a multi-scale three-dimensional conductive network that reduces contact resistance, improves signal stability, and enhances low-pressure response [39,40,41,42].

In addition to material selection, microstructure design is an important strategy for improving flexible strain-sensing performance. Wrinkles, pores, grids, and microcracks can regulate local stress distribution and conductive-pathway evolution, among which microcrack structures have attracted considerable attention because of their high sensitivity to small deformations [43,44,45]. In the initial state, pre-induced microcracks place parts of the conductive network near a critical conduction state. Under tensile deformation, crack opening and increased separation between conductive junctions reduce the number of effective pathways and increase resistance; upon unloading, elastic recovery promotes partial reconnection of the conductive network [46,47,48]. Combining a CNT/graphene composite conductive network with pre-induced microcracks is therefore expected to enhance deformation sensitivity while maintaining conductive continuity and repeatability over a broad strain range [49,50].

Based on the above analysis, this paper proposes a CNT/graphene–TPU nanofiber sandwich-structured flexible strain sensor with pre-induced microcracks. First, a TPU nanofiber membrane was prepared by electrospinning to construct a flexible porous support layer. Then, a CNT/graphene composite conductive layer was deposited by spray-coating. Pre-stretching was subsequently used to induce microcracks in the conductive layer, enabling resistance modulation through crack evolution and conductive-path reconstruction during deformation. Finally, a sandwich structure was constructed using TPU encapsulation films to improve device integrity and wearability. This article discusses material morphology, structural composition, the conductive mechanism, tensile strain response, response/recovery behavior, cycling stability, and qualitative human-motion monitoring applications, aiming to provide an experimental basis for the structural design and application of flexible strain sensors.

## 2. Experimental Section

### 2.1. Materials

The graphene (monolayer graphene aqueous solution, 1 mg/mL) and CNTs (carbon nanotubes dispersion, 1 mg/mL) used in this experiment were purchased from Shenzhen Suiheng Technology Co., Ltd. (Shenzhen, China); TPU pellets were purchased from Guangyuan Plasticization Co., Ltd. (Guangdong, China); Tetrahydrofuran (THF) was purchased from McLean Chemical Reagent Co., Ltd. (Shanghai, China); *N*, *N*-dimethylformamide (DMF, AR ≥ 99.5%) and anhydrous ethanol (EtOH) were purchased from Aladdin Chemical Reagent Co., Ltd.(Shanghai, China). All reagents were used without further purification.

### 2.2. Construction of Microcrack Structure

**Figure 1:**
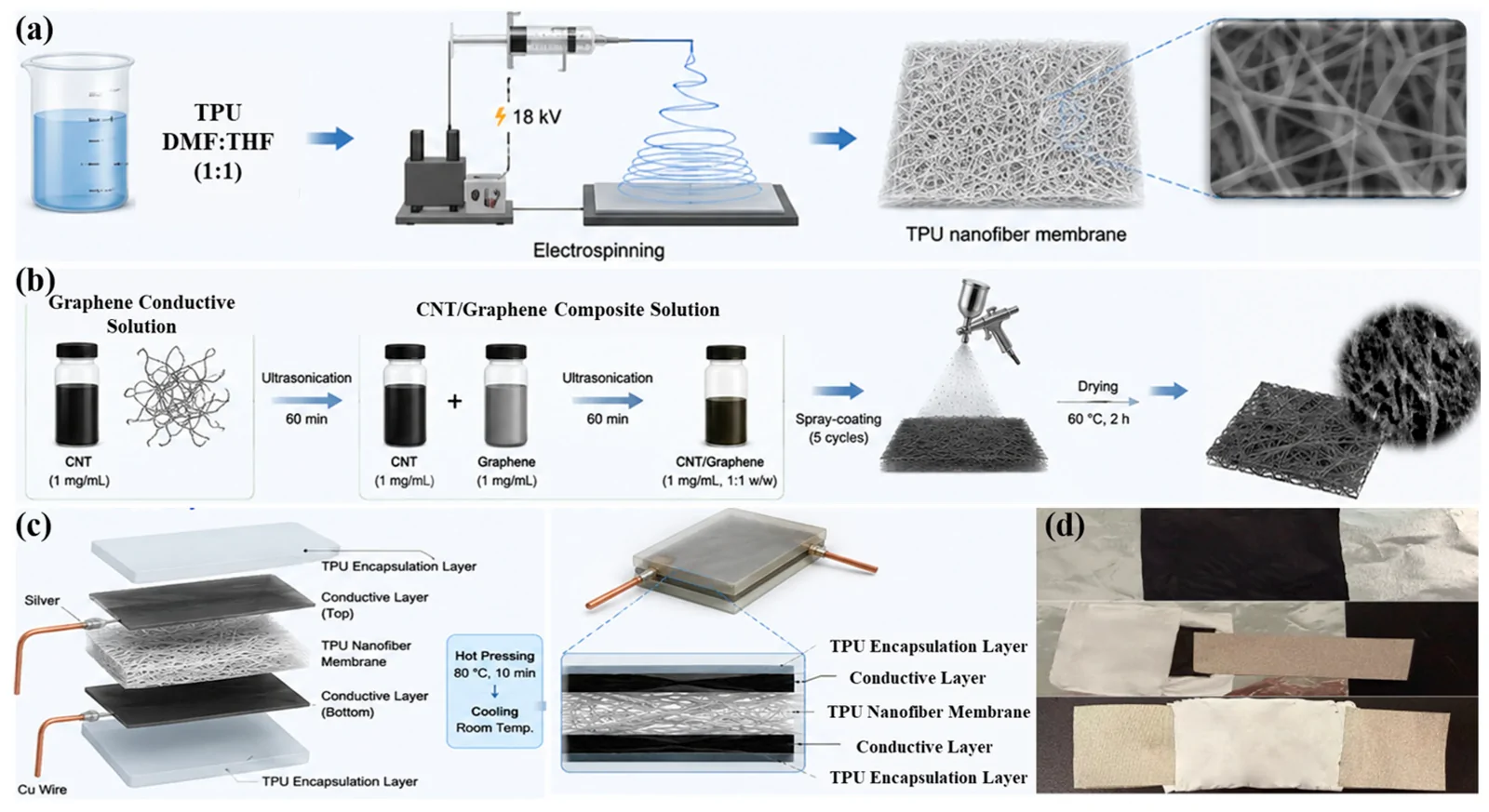
Schematic illustration of the fabrication process for the CNT/graphene–TPU flexible strain sensor. (**a**) Preparation of the TPU nanofiber membrane by electrospinning; (**b**) preparation, sonication, spray-coating, and drying of the CNT/graphene conductive layer; (**c**) stacking and hot-pressing of the sandwich structure; and (**d**) the assembled device.

TPU nanofiber membranes were prepared by electrospinning and used as the flexible supporting substrate of the sensor, as schematically shown in Figure 1a. TPU pellets were dissolved in a DMF/THF mixed solvent at a TPU mass fraction of 18 wt%, with DMF:THF = 1:1, and shaken at room temperature for 180 min until a uniform transparent solution was formed. The solution was then left to stand for 60 min to remove internal bubbles. The spinning solution was transferred into a 5 mL syringe fitted with a 23 G needle and mounted on a syringe pump. The electrospinning parameters were a voltage of 18 kV, a receiving distance of 15 cm, a flow rate of 0.8 mL/h, and a collector rotation speed of 50 rpm, with aluminum foil used as the collection substrate. After spinning, a TPU nanofiber membrane was obtained, whose interwoven fibers formed a three-dimensional porous network that provided a structural basis for conductive-filler loading and deformation transfer. The electrospinning equipment used was a laboratory-made device. The ultrasonic parameters for the conductive composite solution dispersion were as follows: frequency—20 kHz; actual ultrasonic power—approximately 80 W; temperature—20–25 °C.

The conductive layer was constructed by spray-coating, as shown in Figure 1b. For the CNT/graphene composite conductive layer, the CNT dispersion was mixed with the graphene dispersion at a mass ratio of 1:1 and sonicated for 60 min to obtain a uniform composite dispersion. The archived laboratory record does not contain the ultrasonic power, frequency, or bath temperature, and these values were therefore not retrospectively estimated. The dispersion was spray-coated five times and dried at 60 °C for 2 h before being deposited on the TPU nanofiber membrane. In the composite conductive layer, one-dimensional CNTs can bridge gaps between graphene domains, while graphene provides planar conductive contacts. This hybrid architecture is expected to improve conductive-path continuity and electromechanical response stability.

After deposition of the conductive layer, a pre-tensile strain of 5–15% was applied to induce microcracks along the loading direction. After a short holding period, the strain was released and the film returned to its original length, leaving a stable crack network. Crack density, crack width, and crack spacing were not quantitatively measured or used as calibrated structural variables in this study; accordingly, no claim of precise crack controllability is made. The pre-induced crack network places the conductive layer in a deformation-sensitive state in which tensile loading opens cracks and increases junction separation, whereas unloading promotes reconnection of conductive pathways.

The prepared conductive-layer film was stacked with TPU encapsulation films, as shown in Figure 1c, to form a sandwich structure of TPU encapsulation layer/conductive layer/TPU nanofiber layer/conductive layer/TPU encapsulation layer. Conductive copper wires were used as external electrodes and fixed with conductive tape. The assembly was then hot-pressed at 80 °C for 10 min to bond the encapsulation layers to the conductive layers. The sandwich structure protects the conductive layer from external friction and sweat and improves interfacial integrity, allowing external deformation to be transferred to the sensitive layer while enhancing device stability and wearable applicability.

### 2.3. Characterization

The surface morphology of the TPU nanofiber membrane and CNT/graphene–TPU composite layer was observed using field-emission scanning electron microscopy (FE-SEM, HITACHI S-3400N, Tokyo, Japan). The archived imaging record does not retain the accelerating voltage, working distance, detector setting, or sputter-coating condition; therefore, the SEM images are interpreted qualitatively and are not used for quantitative crack metrology. Electrical resistance was measured in a two-terminal configuration between the two attached copper-wire electrodes using a digital multimeter (Keysight 34465A, Penang, Malaysia), while tensile deformation was applied using a digital push–pull force gauge (SH-500; range: 50 N; resolution: 0.1 N, Shanghai, China). The quantitative electrical tests comprised current-voltage (I–V) characteristics, tensile strain-resistance response, response/recovery behavior, repeated loading–unloading under prescribed tensile strains, and long-term cycling stability. The wearable tests in Section 4 were qualitative demonstrations: contact area and preload were not standardized, and resistance-time traces were recorded using the same two-terminal electrical readout. The archived record does not retain a verified software sampling interval or acquisition rate for these demonstrations, and no numerical acquisition-rate value was retrospectively reconstructed.

## 3. Results and Discussion

### 3.1. Characterization of CNT/Graphene–TPU Sensor

**Figure 2:**
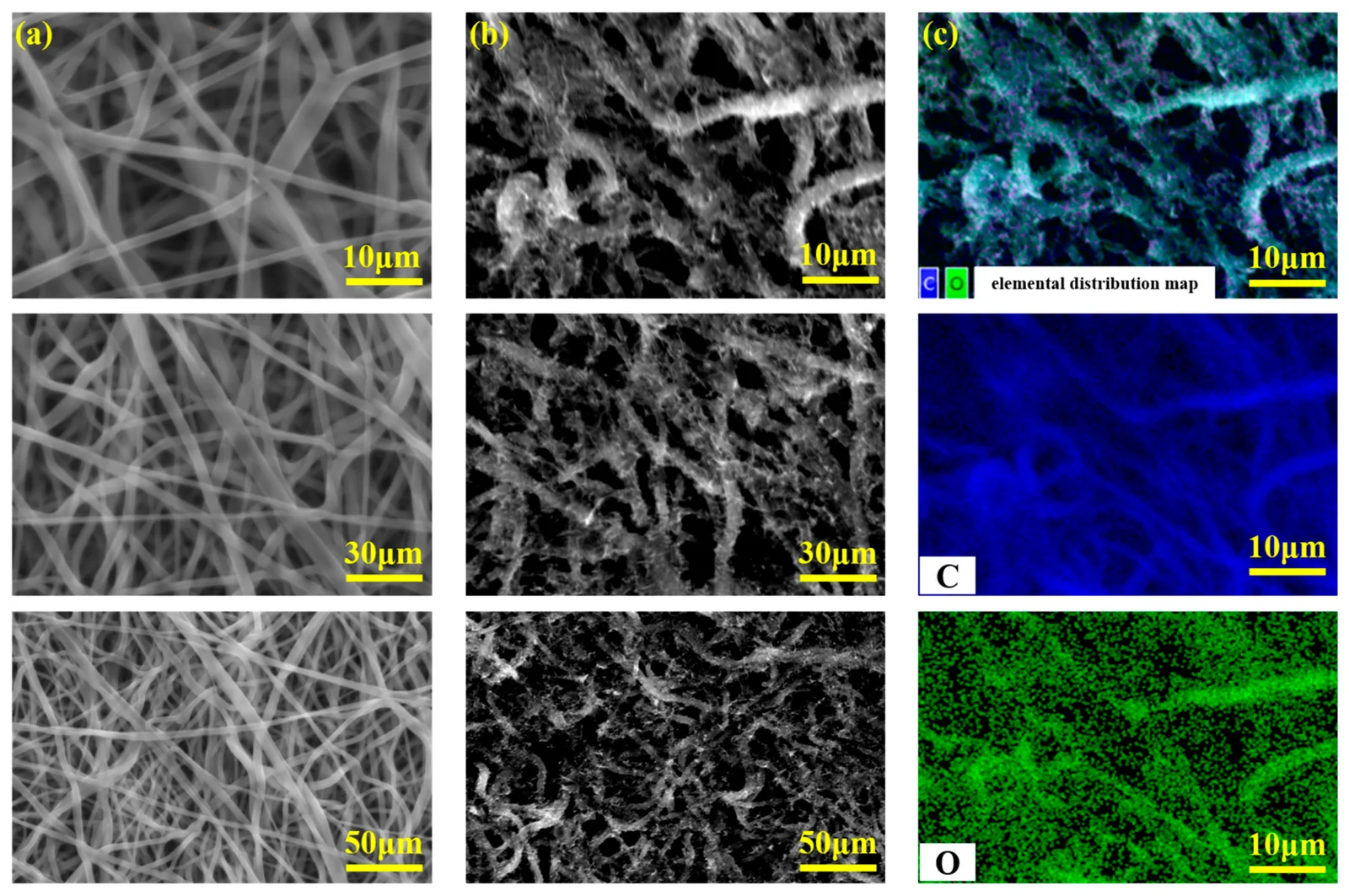
SEM images of (**a**) the TPU nanofiber film and (**b**) the CNT/graphene–TPU thin film, and (**c**) EDS mapping images of the CNT/graphene–TPU thin film.

Figure 2 shows SEM images of the prepared sensor. The TPU nanofiber membrane prepared by electrospinning exhibits a typical three-dimensional interwoven network, with interconnected pores and junctions between fibers, as shown in Figure 2a. This porous morphology increases the available surface area for deposition of conductive fillers and provides a local deformation and recovery space during mechanical loading. Similar electrospun TPU fiber networks have been used as compliant supports in flexible sensing systems because their porous architecture can accommodate repeated deformation [31,32].

Figure 2b shows the TPU fibrous substrate after deposition of the CNT/graphene conductive dispersion. The SEM images show a roughened, interconnected coating on the fiber surfaces and at fiber junctions, consistent with the formation of a hybrid conductive network. The EDS maps in Figure 2c show broadly distributed carbon and oxygen signals across the observed region. Because TPU itself contains both carbon and oxygen, these EDS maps cannot selectively distinguish the CNT/graphene coating from the polymer substrate; they are therefore presented only as elemental-distribution information and not as proof of uniform CNT/graphene impregnation. The morphology-based interpretation of a CNT/graphene hybrid conductive network is consistent with previously reported CNT/graphene systems [36].

### 3.2. Mechanical Adaptability and Interface Stability

Flexible strain sensors used in wearable environments undergo bending, stretching, compression, and friction. The elastic TPU nanofiber network can accommodate repeated deformation and provides a compliant support for the conductive coating. Similar TPU-based fibrous substrates have been reported to improve mechanical adaptability in wearable sensing systems [31,32].

The sandwich encapsulation structure further improves device integrity by constraining lateral slip of the conductive layer during bending and stretching and by reducing direct exposure of the conductive network to sweat, dust, and mechanical friction. The graphene domains can provide distributed conductive contacts, while CNTs can bridge neighboring domains and help maintain conductive continuity during deformation [39,41]. These structural features provide a basis for the stable deformation response evaluated below; however, no independent interfacial-strength test was performed in this study.

### 3.3. Sensing Mechanism of Microcrack-Regulated Conductive Network

The piezoresistive response of the CNT/graphene–TPU strain sensor is attributed to the combined effects of conductive-contact rearrangement, microcrack evolution, and tunneling-distance variation. In the initial state, CNTs and graphene form an interconnected but non-dense conductive network on the TPU fibers, while pre-induced microcracks introduce locally weak conductive connections. Under tensile strain, crack opening and increased spacing between CNT/graphene junctions reduce the number of effective conductive pathways and increase the overall resistance. During unloading, elastic recovery of the TPU substrate promotes partial closure of the cracks and reconnection of conductive contacts. This mechanism is consistent with previously reported crack-based strain sensors [43,48,49].

As tensile strain increases, progressive microcrack opening and junction separation can drive the conductive network closer to a percolation transition, producing a nonlinear increase in resistance and a higher apparent gauge factor at larger strains. At the same time, CNTs bridges help preserve electrical continuity so that the signal remains recoverable during unloading and repeated cycling. The combination of crack-mediated resistance amplification and a hybrid CNT/graphene network therefore provides a plausible explanation for the piecewise strain sensitivity observed in Section 3.4 [43,48,49,51].

The sensitivity of a resistive strain sensor is commonly represented by the gauge factor (GF), which is defined as [51]

(1) $$GF=\frac{R-R_{0}}{R_{0}\varepsilon }$$

where GF is the gauge factor; *R*_0_ is the initial resistance of the sensor at zero strain; *R* − *R*_0_ is the resistance change caused by deformation; and *ε* is the tensile strain of the sensitive element. Strain is defined as

(2) $$\varepsilon =\frac{∆L}{L}$$

where *ε* is dimensionless, Δ*L* is the change in length, and *L* is the original length. In Equation (1), Δ*R*/*R*_0_ is the relative resistance change and is also dimensionless.

### 3.4. Tensile Strain Sensing Performance

**Figure 3:**
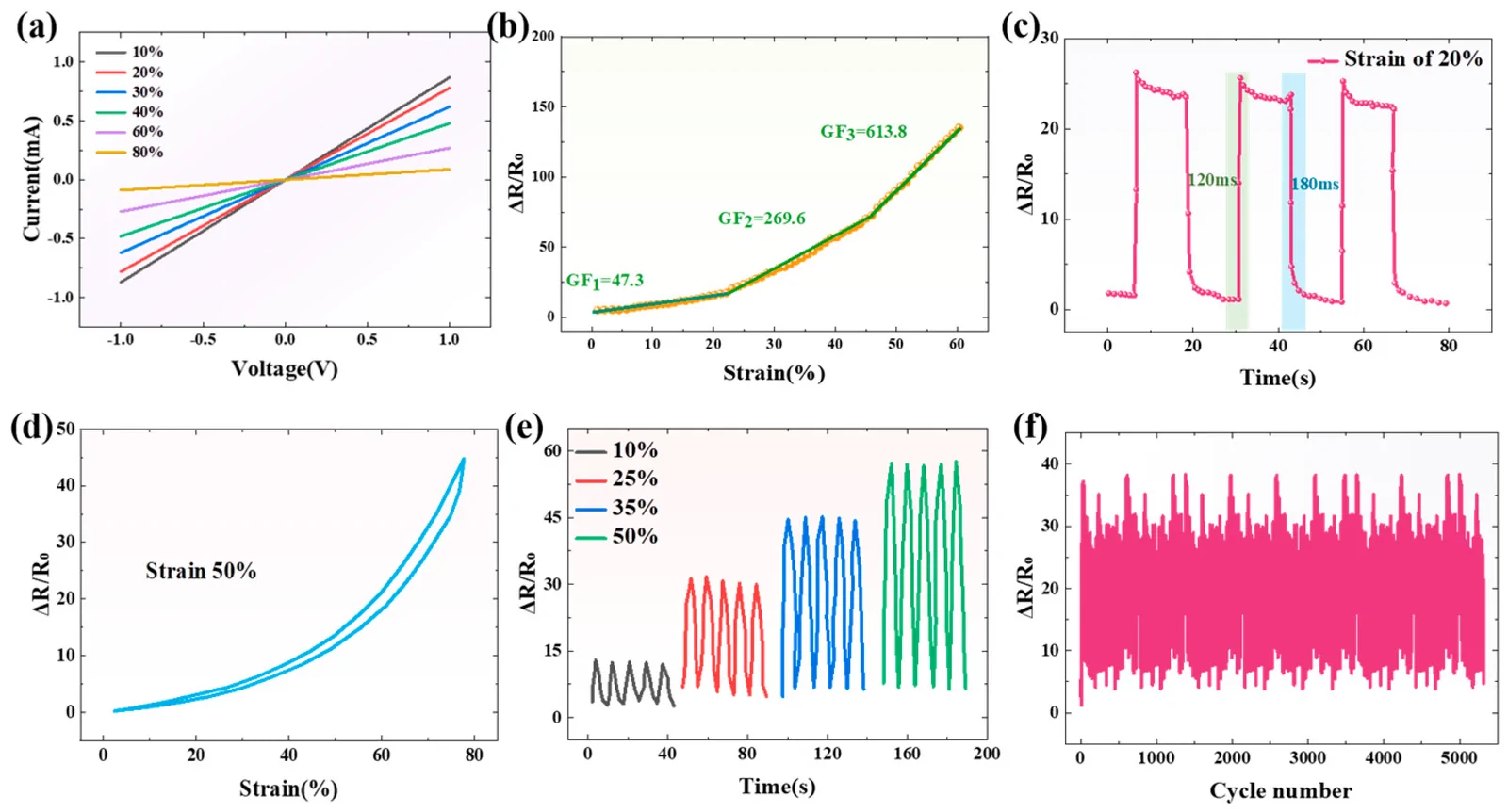
(**a**) I–V curves under tensile strains of 10–80%; (**b**) relative resistance change as a function of strain and the corresponding piecewise gauge factors; (**c**) response and recovery under repeated loading–unloading at 20% strain; (**d**) loading–unloading hysteresis curve up to a maximum strain of 50%; (**e**) repeated tensile responses at different strain levels; and (**f**) electrical response over 5000 loading–unloading cycles at 20% strain.

To evaluate electrical behavior under tensile deformation, the current–voltage (I–V) characteristics were first measured at different strain states. The two ends of the sensor were fixed in the stretching fixture, and quasi-static tensile strains from 10% to 80% were applied while the electrical response was recorded using the two-terminal configuration described in Section 2.3. As shown in Figure 3a, the I–V relationship remains approximately linear over the tested strain states, indicating stable electrode contact and resistive readout behavior.

Using the GF definition in Equation (1), the relative resistance change Δ*R*/*R*_0_ was plotted against tensile strain *ε* in Figure 3b. Three approximate linear regions are observed. Over 0–20% strain, GF is approximately 47.3; over 20–45% strain, GF increases to approximately 269.6; and over 45–60% strain, GF reaches approximately 613.8. The increase in GF with strain is consistent with the progressive disruption of conductive pathways and microcrack opening in crack-based conductive networks [43,48,49,51].

As shown in Figure 3c, the CNT/graphene–TPU sensor exhibits response and recovery times of approximately 120 and 180 ms, respectively, under repeated tensile loading at 20% strain. This response speed supports the detection of transient mechanical deformations and time-varying physiological or motion-related signals [52].

In addition, as shown in Figure 3d, the sensor exhibits a hysteresis loop during loading and unloading to a maximum strain of 50%. At low strain, the loading and unloading traces are relatively close, whereas the hysteresis becomes more apparent at larger strain. This behavior is attributable in part to the viscoelastic recovery of the TPU substrate and delayed reconstruction of the conductive network, which are common considerations in polymer-based strain sensors [51,52].

Under repeated stretching over strains of 10–50%, the relative resistance change Δ*R*/*R*_0_ increased monotonically with strain (Figure 3e), indicating a positive correlation between electrical response and tensile deformation. The repeatable signal levels at each applied strain further indicate stable strain discrimination over the tested range.

Finally, the CNT/graphene–TPU sensor was subjected to 5000 consecutive loading–unloading cycles at 20% tensile strain (Figure 3f). The resistance-response waveform remained observable throughout the cycling test, with no obvious catastrophic signal loss, demonstrating the durability of the electrical response under repeated tensile deformation.

To further place the present design in context, representative flexible strain-sensor architectures are compared in Table 1. Reported approaches include composite conductive networks, sandwich encapsulation, crack engineering, and oriented fibrous substrates. The PPy-CNT/TPU system shows that dual conductive layers can help balance wide-range strain detection and high sensitivity [52], while graphene-based sandwich structures illustrate the role of interfacial integrity in wearable sensing. Oriented nanofiber-MWCNT designs further show that fiber organization and crack evolution can improve sensitivity and cyclic response [53]. The present CNT/graphene–TPU device combines a porous TPU support, a hybrid CNTs/graphene conductive network, pre-induced microcracks, and sandwich encapsulation to obtain a broad tensile strain response with piecewise GF values.

## 4. Sensor Applications

The ultimate value of wearable flexible strain sensors lies in their real-time recognition of human motion and physiological signals. This study affixed CNT/graphene–TPU sensors to the finger, pulse, elbow, plantar region, and a water cup, and tested their response characteristics during different human movements. Owing to the flexible TPU encapsulation layer, the device can conform well to skin or joint surface without causing significant foreign-body sensation during bending and compression, which facilitates continuous acquisition of human motion signals.

**Figure 4:**
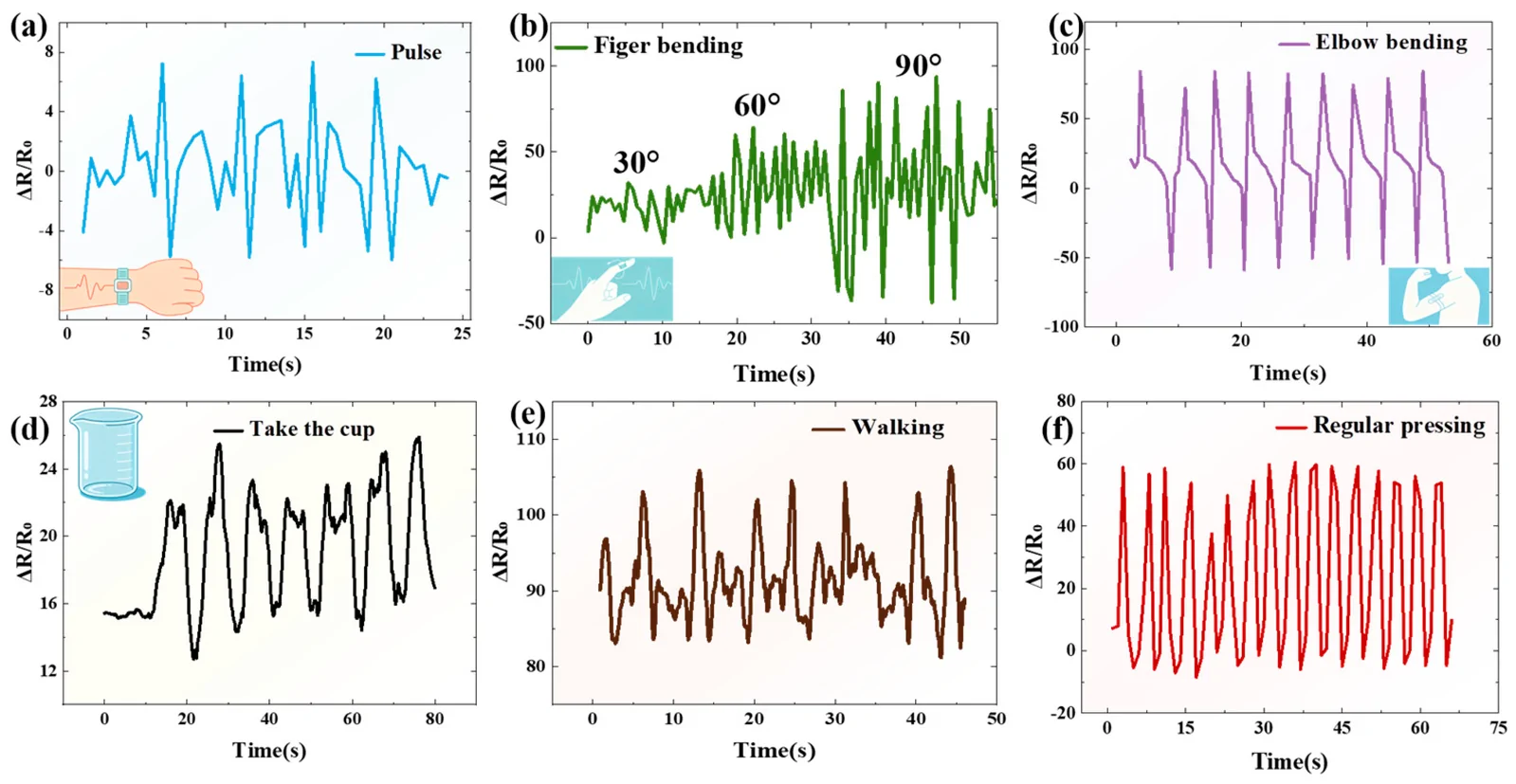
(**a**) Pulse detection, (**b**) finger-bending, (**c**) elbow-bending, (**d**) holding an empty beaker, (**e**) walking, and (**f**) regular pressing.

In pulse monitoring, the amplitude of the human pulse signal is small and the frequency is high, requiring high low-pressure sensitivity and fast response speed of the sensor. As shown in Figure 4a, the sensor exhibits high sensitivity in the low-pressure regime and can capture small pressure changes caused by periodic pulsation of blood vessels, providing the possibility for extracting pulse frequency, waveform features, and health status parameters in the future. In the finger-bending test, as the bending angle increases, the compression and stretching effects on the sensor at the joint intensify, and the relative resistance change amplitude increases accordingly, as shown in Figure 4b. The output curve presents clear, distinguishable stepped change. This indicates that the sensor can recognize finger movements of different amplitudes, which can be used for gesture recognition, rehabilitation training evaluation, and human–computer interaction input. For monitoring human joint movements, the sensor can accurately respond to continuous flexion and extension of the elbow (Figure 4c), and its relative resistance changes are highly synchronized with the amplitude and frequency of the movement, with rapid response and good repeatability, indicating its reliability in human motion posture recognition and rehabilitation training monitoring. Figure 4d shows the signal feedback curve of the sensor when grasping and releasing the beaker. The sensor is placed tightly in the palm of the experimenter’s hand, and when the water cup is lifted, the sensor is compressed to produce an upward signal. When the water cup is placed down, the response signal decreases. In gait recognition testing, plantar pressure undergoes periodic changes during standing, walking, and foot-lifting. As shown in Figure 4e, the sensor can output pressure signals with significant peak differences, indicating that it can be used for gait analysis, motion monitoring, and smart insoles. Finally, a regular press was applied to the sensor, and Figure 4f further demonstrates the sensor’s clear resolution for tactile stimuli in tapping scenarios.

Overall, the CNT/graphene–TPU sensor produced stable and distinguishable resistance-time patterns across several wearable deformation scenarios, supporting its potential for motion recognition and physiological-signal monitoring. Future work may combine multi-channel arrays, standardized mechanical loading, wireless acquisition, and algorithm-based classification to enable quantitative multi-point deformation mapping and automated activity recognition.

## 5. Conclusions

A sandwich-structured CNT/graphene–TPU nanofiber strain sensor was fabricated using electrospinning, spray-coating, pre-stretching, and hot-pressing. The study evaluated the relationship between the hybrid conductive-network structure and tensile strain-sensing performance, while TPU sandwich encapsulation was used to improve device integrity and support repeated deformation.

The CNT/graphene composite coating forms an interconnected conductive network on the TPU fibrous substrate, while pre-induced microcracks provide deformation-sensitive changes in conductive-path continuity. Under tensile loading, the sensor exhibited piecewise gauge factors of approximately 47.3, 269.6, and 613.8 over strain ranges of 0–20%, 20–45%, and 45–60%, respectively, together with response/recovery times of approximately 120/180 ms and an observable electrical response over 5000 loading–unloading cycles at 20% strain. Qualitative demonstrations involving pulse, finger-bending, elbow motion, grasping, walking, and repeated pressing produced distinguishable resistance-time signals, indicating potential for wearable deformation monitoring. No normal-pressure sensitivity or pressure range is claimed because a normal-pressure calibration was not performed.

In summary, a hybrid conductive-network design, pre-induced microcracks, and flexible sandwich encapsulation are effective strategies for obtaining a sensitive and durable strain-dependent electrical response. Future work should quantify crack morphology, standardize specimen geometry and mechanical loading, report complete equipment and acquisition parameters, and perform calibrated normal-force tests if pressure-sensing performance is to be evaluated. Integration with flexible arrays and wireless acquisition may further support multimodal deformation monitoring and practical wearable applications.

## Figures and Tables

**Table 1:** Comparison of sensing performance between this work and previously reported flexible sensors.

Sensor Name	Key Design	Sensitivity	Stability	Ref.
PPy-CNT/TPU	Electrospinning TPU + double conductive layer	GF = 140.9 (0–180%); GF = 438.7 (180–400%)	Response/recovery: 40/69 ms; 6000 cycles	[52]
Ecoflex/graphene/Ecoflex	Graphene sandwich structure	GF = 27.46 (0.5–30%); GF = 132.83 (30–50%)	Response time: 111 ms	[4]
TPU/PAN-MWCNT	Oriented nanofibers + crack design + forked electrode	GF = 29–46 (3.3–43.3%)	1000 cycles	[53]
CNT/graphene–TPU	CNTs/graphene composite network + microcracks + TPU sandwich encapsulation	GF = 47.3 (0–20%); GF = 269.6 (20–45%); GF = 613.8 (45–60%)	Response/recovery: approximately 120/180 ms; >90% signal retention after 5000 cycles	This work

Note: Values in parentheses indicate the strain range (%).

## Data Availability

The original contributions presented in this study are included in the article. Further inquiries can be directed to the corresponding author.

## Abbreviations

The following abbreviations are used in this manuscript:

CNT	Carbon nanotube(s)
TPU	Thermoplastic polyurethane
GF	Gauge factor
FE-SEM	Field-emission scanning electron microscopy
EDS	Energy-dispersive X-ray spectroscopy
DMF	*N*,*N*-dimethylformamide
THF	Tetrahydrofuran
EtOH	Ethanol
I–V	Current-voltage
UV	Ultraviolet
